# Refining Clinical Quantification of Depth of Suppression in Amblyopia through Synoptophore Measurement

**DOI:** 10.3390/life13091900

**Published:** 2023-09-12

**Authors:** Maureen D. Plaumann, Krista L. Roberts, Wei Wei, Chao Han, Teng Leng Ooi

**Affiliations:** College of Optometry, The Ohio State University, Columbus, OH 43210, USA; mdplaumann@uh.edu (M.D.P.);

**Keywords:** suppression, Synoptophore, illuminance, amblyopia, stereopsis

## Abstract

Background: Amblyopia is associated with unbalanced suppression between the two eyes. Existing clinical measures of suppression, such as the Worth 4 Dot test, provide qualitative information about suppression but cannot precisely quantify it. The Synoptophore, a well-established instrument in binocular vision clinics, has historically been used to gauge suppression qualitatively as well but has the capability to quantify suppression. We extended the capability of the Synoptophore through the development of a systematic protocol of illumination manipulation to quantify suppression in amblyopia. Methods: Twenty-six previously treated adult amblyopes underwent our protocol on the Synoptophore to measure the illumination balance needed to obtain fusion responses. Separately, these same amblyopes were tested with Worth 4 Dot as it is classically performed in the United States, utilizing different test distances and room illuminations to qualify the suppression response. Results: Smaller, more central targets revealed larger magnitudes of suppression for both the Synoptophore and Worth 4 Dot tests (Synoptophore: χ^2^_5,26_ = 25.538, *p* < 0.001; Worth 4 Dot: χ^2^_3,26_ = 39.020, *p* < 0.001). There was a significant correlation between the two tests for depth of suppression measurements (*r_Τ_* > 0.345, *p* < 0.036), with more sensitivity measured by the Synoptophore, as suppression could be graded on a quantitative scale. Strabismic amblyopes demonstrated more suppression than non-strabismic amblyopes (*z* > 2.410, *p* < 0.016). Additionally, depth of suppression was correlated with interocular difference in both visual acuity (*r_Τ_* = 0.604, *p* < 0.001) and stereoacuity (*r_Τ_* = 0.488, *p* = 0.001). Conclusions: We extended the utility of the Synoptophore by measuring its illuminance outputs and developing a suppression testing protocol that compared favorably with Worth 4 Dot (clinic standard) while improving upon the latter through more sensitive quantification of suppression.

## 1. Introduction

Amblyopia in humans is associated with decreased visual functions due to abnormal binocular interaction during the development of the visual system [1], and it affects approximately 2% of the population [2]. Often, these decreased visual functions are discussed in terms of monocular visual acuity deficits, though other deficits exist in amblyopia that are both monocular and binocular in nature. Binocular deficits include excessive interocular suppression of the amblyopic eye and reduced stereoacuity [1,3,4,5,6,7,8,9]. The importance of addressing suppression to understand amblyopia has received much attention in the recent literature. Psychophysical studies have shown that implementing binocular perceptual learning protocols to reduce the suppression of the amblyopic eye resulted in improved visual functions [10,11]. Although suppression is often measured clinically, its outcome is usually recorded in a binary manner (yes/no). This is because the tests that evaluate suppression do not readily lend themselves to precise measurements of suppression magnitudes. A common clinical test for suppression is the Worth 4 Dot test [12], but it lacks precision for quantifying the amount of suppression. Often, clinicians use ambient lighting as a measure of suppression, noting that if a patient suppresses in both a bright and dim room, the suppression is deep, while if he/she only suppresses in a bright room, the suppression is shallow [8]. Even when a filter is used in conjunction with the Worth 4 Dot test, the dissociative nature of the red green filters (that create the dichoptic viewing conditions necessary to evaluate suppression) can give rise to artifacts in fusion responses [13].

More sensitive measurements of suppression using standard psychophysical methods exist within the laboratory setting. These include tests with global motion and spatial pattern stimuli [14], with binocular rivalry stimulus [10,15,16,17,18], and with binocular phase combination stimulus [19,20,21]. While these tests provide the precise quantification of suppression depth, they are not readily accessible to clinicians because they require an elaborate hardware and software setup. Thus, a gap remains as to how clinicians can precisely quantify suppression depth in a manner akin to the stricter laboratory measures. Given the important role of suppression in amblyopia, it is more imperative now for clinicians to quantify suppression, in addition to visual acuity. Knowing the extent of suppression provides a useful index for diagnosing the severity of amblyopia and for monitoring the recovery progress during amblyopia therapy. Other researchers have recommended procedures that can be completed clinically to quantify suppression using different computer-based technologies [22,23]. There is also a novel eye movement analysis that provides insight into stereopsis, binocular summation, and suppression through the measurement of the Ocular Following Responses that negates the need for subjective responses from patients [24,25]. While the outcomes from these studies provide promising results for the field of amblyopia, they are currently limited in use and require specialized equipment.

We propose that the Synoptophore could be used as the instrument to bridge the gap between laboratory and clinic. The earliest Synoptophore was constructed by William Ettles in 1912. It was based on a haploscopic design of the Amblyoscope invented by Claud Worth and constructed by AW Haws in 1895 and later modified by Nelson Black in 1906 [26]. Worth proposed three sequential levels of binocular visual abilities, which he referred to as first, second, and third degrees of fusional abilities (to be described more in Methods) [12]. This concept of three degrees of fusion lays the foundation for clinical testing with the Synoptophore. Today, the Synoptophore is a commercially available instrument present in many Binocular Vision clinics. The Synoptophore does not use dissociative filters, providing for a more naturalistic viewing that may better gauge how patients operate binocularly in their everyday world [27]. It also provides for the independent manipulation of each eye’s stimulus illumination, whereby the amblyopic eye’s target intensity can be boosted, while the fellow eye’s target intensity is diminished via a rheostat mechanism that controls the brightness of the light bulbs used to illuminate the dichoptic targets. Performing this allows the clinician to balance the strength of the two eyes to obtain an estimate of the depth of suppression of the amblyopic (weak) eye, in a manner similar to conventional psychophysical measurements [15,16,28,29,30,31].

While manipulation of the instrument’s rheostats has been used for decades [32], a systematic protocol has not been implemented nor have the illumination settings been published to provide meaningful quantification of suppression (Haag-Streit UK was unable to provide us with the illuminance values (personal communication)). Thus, in terms of illuminance control, the Synoptophore measures provide a little more information than the previously mentioned Worth 4 Dot. To address this deficiency and extend the utility of the Synoptophore, the current study measured the illuminance levels of the Synoptophore at the various rotatory knob settings. This allowed us to precisely quantify the depth of suppression, thus making the measurement more comparable with laboratory measurements of suppression. The ability to quantify suppression also permitted us to measure the magnitude of suppression with Worth’s first- and second-degree fusional targets. In fact, the concept-driven design of the Synoptophore targets, which allows for the classification of successive levels of binocular fusion abilities, provides a clear advantage for quantifying suppression over the Worth 4 Dot test, which is considered a second-degree target with very rudimentary fuse-able contours. Additionally, the commercially manufactured slides used for the present study are printed in a variety of sizes that allows for testing the same target features at different visual angles while maintaining a set viewing distance. Worth 4 Dot is typically tested at different distances to alter the visual angle of the target, making it difficult to precisely control for accommodation, eye alignment, and the visual angle itself. The objective of our study was to design a protocol for measuring suppression in amblyopia with the commercially manufactured Synoptophore and corresponding slides. This would allow for clinicians to measure their patients’ suppression in more precise detail, using equipment they already have available for testing. To provide reassurance that our protocol is suitable for clinical needs, we set out to validate our protocol through comparison to the current clinical standard of Worth 4 Dot. Separately, we tested our subjects’ visual acuity and stereopsis to compare with the suppression magnitudes obtained with the Synoptophore.

## 2. Methods

The research reported herein conformed with the tenets of the Declaration of Helsinki and was approved by The Ohio State University’s Institutional Review Board. Subjects provided written informed consent prior to study participation.

### 2.1. Participants

Twenty-six adult subjects participated in our study, all of whom had been previously treated for amblyopia with varying levels of compliance and success. The average age of the subjects was 27.5 ± 5.5 years, and 65% were female. For simplicity, we refer to our subjects as amblyopic given their history and current binocular deficiencies. Additionally, to be considered for analyses, subjects had to have a best-corrected interocular difference in visual acuity of 0.2 logMAR or more (visual acuity testing is described in further detail below). All testing was conducted with subjects wearing lenses (either habitual eyeglasses or trial frames) that corrected for their anisometropia, as determined by cycloplegic retinoscopy, within 0.50 D. Please refer to Table 1 for details of each subject. 

We classified amblyopia based upon suspected origin. Subjects without a constant unilateral strabismus whose amblyopia was caused by anisometropia were categorized as “Aniso,” even if an intermittent strabismus was also present, which occurred at the near test viewing distance in a few subjects. Subjects with a constant unilateral strabismus were grouped into the “strabismic” amblyopia group, even if anisometropia was also present. No subjects were included with coexisting ocular pathology.

### 2.2. Synoptophore

#### 2.2.1. Apparatus

**Figure 1:**
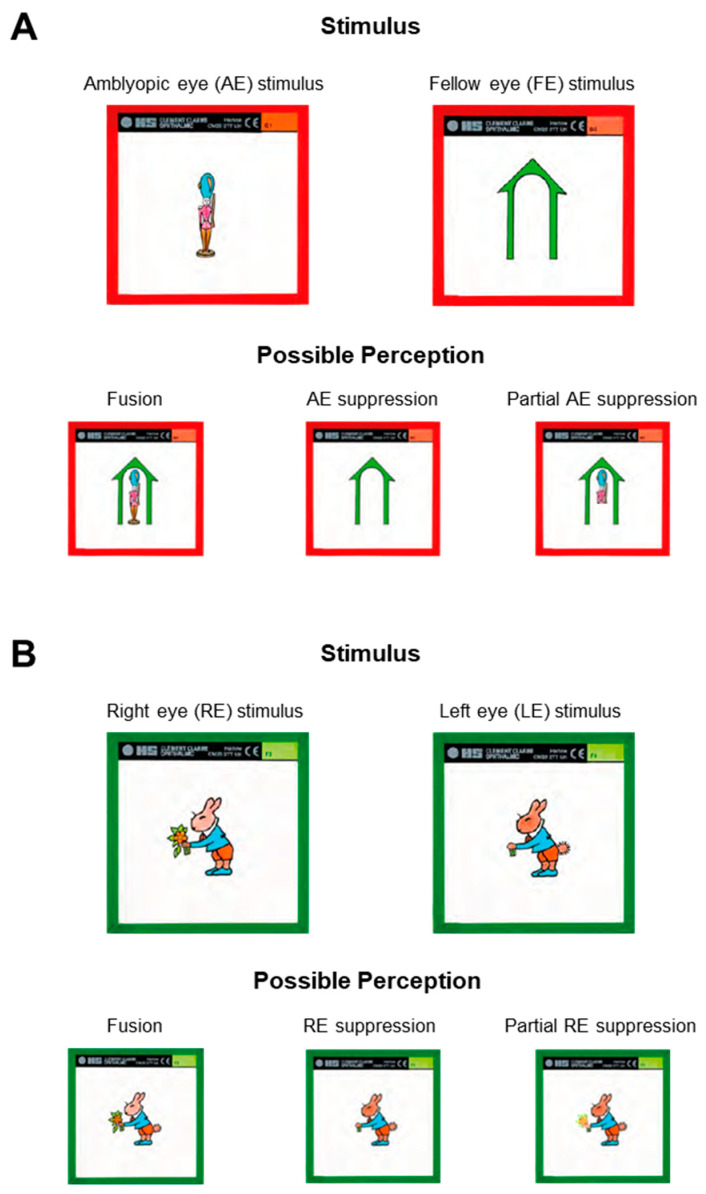
First-degree (**A**) and second-degree (**B**) Synoptophore targets. The stimulus for each target type is presented above its possible perceptions. A fusion response (labeled as “fusion” for both types of targets) includes all features from both eyes; a total suppression response (labeled as “AE suppression” for first-degree target and “RE suppression” for second-degree target) represents only features from the fellow eye of a right eye amblyope; a partial suppression response (labeled as “Partial AE suppression” for first-degree target and “Partial RE suppression” for second-degree target) includes some features from the amblyopic eye, but they may be patchwork, illustrated with soldier missing legs, or faded, illustrated by the rabbits faded flower.

**Figure 2:**
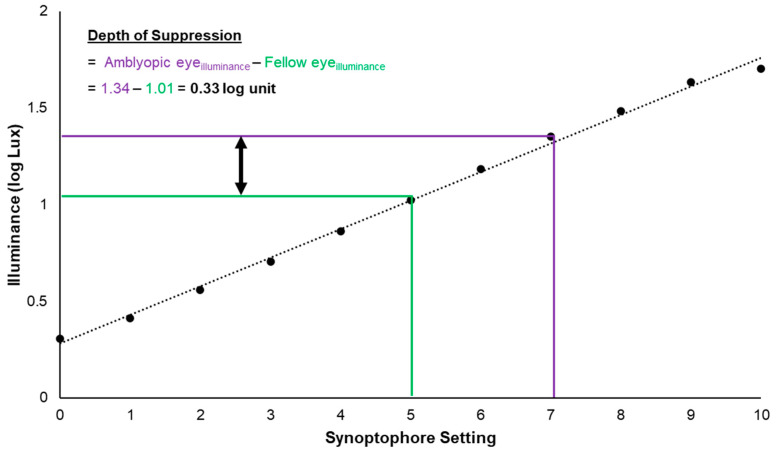
Synoptophore illuminance and sample depth of suppression calculation. The black dots represent the average illuminance output for the Synoptophore and reveal a log-linear relationship as a function of the rheostat setting. To calculate the depth of suppression, the illuminance for the fellow eye is subtracted from the illuminance of the amblyopic eye. In the figure, the fellow eye illuminance (green line) corresponded to Synoptophore setting 5, and the amblyopic eye illuminance (purple line) corresponded to Synoptophore setting 7, when the subject reported the best balance. These Synoptophore settings correspond to 1.01 log Lux and 1.34 log Lux, respectively, where the depth of suppression is the difference between the two settings (0.33 log Lux).

Testing of suppression was performed on the Haag-Streit Synoptophore, 2001 model (Haag-Streit, Koniz, Switzerland). The Synoptophore has two separate slide mounts that are projected to each eye through separate moveable tubes, utilizing a mirror at the bend of the tube and a + 6.50 DS lens to simulate distance viewing. Each target slide was illuminated by the supplied Eveready Clear Capsule G4 20 W Halogen Bulb (Energizer Holdings, St. Louis, MO, USA) that was controlled by a rheostat ranging from 0 to 10, with markings for every integer. A T-10MA Illuminance Meter (Konica Minolta, Ramsey, NJ, USA) was used to measure the radiometric brightness of the Synoptophore at each integer setting (0–10, 11 settings total) through each target slide (described below with examples depicted in Figure 1). An overall log-linear progression of illumination was found in between settings, such that each increase in a setting on the rheostat resulted in an approximately 0.15 log unit increase in brightness. This log-linear relationship is depicted in Figure 2, where a significant correlation (*r_P_* = 0.997, *p* = 0.001) exists between Synoptophore setting and illuminance. 

#### 2.2.2. Stimuli

Target slides for our study were commercially made by Haag-Streit and fell into the simultaneous perception and flat fusion categories of Worth’s three fusion levels. The simultaneous perception slides, referred to throughout the rest of the paper as first-degree targets, pictured in Figure 1A, were a pair of dissimilar objects (a soldier and a house). The flat fusion slides, referred to throughout the rest of the paper as second-degree targets, pictured in Figure 1B, were a pair of similar objects (to promote fusion) tagged with different features (serving as suppression checks) presented to each eye (a rabbit with flowers and a rabbit with a tail). These particular stimuli were chosen as they are manufactured in a variety of sizes. The angular sizes of the three soldiers were, respectively, 12 × 2.5°, 7.5 × 1.5°, and 2.5 × 0.5°, and the angular sizes of the three rabbits were, respectively, 11 × 11°, 7 × 7°, and 2.5 × 2.5°. 

### 2.3. Measurement of Depth of Suppression with the Synoptophore

Depth of suppression was measured when subjects reported simultaneous perception with the first-degree targets or fusion with the second-degree targets. To achieve this, the dichoptic targets were initially placed at the subject’s objective angle (determined as the position where alternating illumination resulted in no movement of either eye) and then fine-tuned with some subjective input (subjects reported whether images were diplopic at the objective angle, and adjustments were made until single vision was obtained). A simultaneous perception response was determined when the subject saw the soldier centered in the house. A flat fusion response was determined when all suppression checks were visible and aligned with second-degree targets (e.g., a single rabbit with a tail holding flowers). Each dichoptic target was initially illuminated with setting “5” on its corresponding rheostat (rotatory knob). Illumination was then altered to find the setting for each eye in which it was easier to “focus on”, “attend to”, or “see with equal ease” features from both eyes’ targets. For example, with the first-degree slides, a subject might report the soldier’s hat was “flickering in and out” during the one setting and appeared “more stable” during another. In this example, the rheostat settings in which the soldier appeared the “most stable” with the least amount of flickering would be used to determine the depth of suppression.

Rheostat manipulation (by the experimenter) was always in the same order. First, the amblyopic eye’s illuminance was increased by integer steps (e.g., 5 to 6, 6 to 7, etc.) where each subject was asked to identify the preferred setting by choosing between the two options presented. Illumination to the amblyopic eye was increased until a higher setting was rejected. Then, the fellow eye’s illuminance was decreased by integer steps (e.g., 5 to 4, 4 to 3, etc.) in the same manner until a lower setting was rejected. Depth of suppression was calculated as the illuminance of the fellow eye subtracted from the illuminance of the amblyopic eye (log Lux _amblyopic eye_ − log Lux _fellow eye_), as demonstrated in Figure 2.

Suppression was measured for each pair of targets, resulting in six measures of depth of suppression for each subject (2 target types × 3 stimulus sizes). Targets were measured in sequential order for all subjects with all first-degree targets measured first, in order from largest to smallest angular sizes, and then all second-degree targets measured in order from largest to smallest angular sizes. This order of testing was chosen as it corresponds to the order typically performed in clinical testing [32].

For both target types, if a subject was unable to achieve the correct fusional response (e.g., soldier centered in the house or rabbit with both flowers and tail), then illumination was increased to 10 for the amblyopic eye and decreased to 0 for the fellow eye. If this still did not result in the perception of the suppressed feature, 0 and 10 were used to calculate the depth of suppression, even though suppression was still present. While arbitrarily assigning “10/0” is an overestimation of the subject’s ability to overcome suppression, we decided to perform this in order to include his/her data in our analysis.

### 2.4. Worth 4 Dot

#### Apparatus and Stimuli

Testing of suppression was performed with the Worth 4 Dot flashlight (Bernell, Mishawaka, IN, USA). This commercially available flashlight has four circular targets arranged in a diamond pattern. The overall height and width of the diamond (from outside edge to outside edge) was 35 mm, while the diameter of each dot was 6 mm. Thus, at 40 cm, the visual angle of the entire diamond subtended approximately 5°, with each dot subtending an angle of approximately 0.859°. These angular sizes decreased to approximately 0.668° and 0.115°, respectively, when the target was placed at 3 m. 

### 2.5. Measurement of Depth of Suppression with the Worth 4 Dot

Depth of suppression was measured in a manner that is consistent with the clinical setting, and in all instances, Worth 4 Dot testing was completed after Synoptophore testing. Subjects were seated and shown the flashlight while wearing red/green glasses with the red lens over the right eye. Starting at a viewing distance of 40 cm with the room fully illuminated, subjects were asked to report the following observations: how many lights they saw, what colors they were, if all lights were of equal brightness, if lights were present the entire time or disappearing at times, and whether any of the lights appeared “cut-off” or had parts missing. If a subject reported unequal brightness, then the fellow eye was occluded, and the subject was asked if the brightness of the remaining lights, viewed by the amblyopic eye, changed. If they got brighter, then our assumption was that unequal brightness during the binocular viewing condition was due to underlying suppression and not uneven filter transmission. Thus, a partial suppression categorization was assigned when subjects reported unequal brightness in all four lights, with the amblyopic eye’s lights dimmer. Other categorizations of partial suppression are reported in Table 2, as well as how no suppression and total suppression were defined.

If a subject reported any other response besides “no suppression,” loose prisms were held over the amblyopic eye to determine if compensating for a subject’s misalignment in free space altered their suppression response. The prism power demonstrated was determined based on the subject’s cover test at that particular viewing distance. If this “corrective” prism caused the subject to experience diplopia when they previously had not, then a different prism was selected to view through based on the subject’s subjective angle from Synoptophore testing. For our analysis, the “best” response was used, e.g., if a subject reported unequal brightness without prism and then equal brightness with, a categorization of “no suppression” was used for that particular testing distance. This prism procedure may not always be adopted in the clinical setting, as some clinicians may only be interested in the habitual viewing condition. However, the prism for motor alignment was indicated to better compare the Worth 4 Dot findings to the Synoptophore, where depth of suppression measures were taken in a manner in which the manifest deviation was compensated.

After completing testing at 40 cm with full room illumination, the lights were turned off, and testing was repeated at 40 cm in dim illumination. The testing distance then changed to 3 m with the lights back on and finally concluded at 3 m with the lights turned off. The testing sequence was the same for all subjects and was completed in the order described here: near/light, near/dark, distance/light, distance/dark. This sequence was chosen as it best matched the sequence tested in the Synoptophore (with larger visual angle targets measured first) and follows the clinical convention for Worth 4 Dot testing, in which room illumination is dimmed following an abnormal response to elicit a potential normal response.

### 2.6. Clinical Measures of Other Visual Functions

Distance visual acuity was measured with a Revised ETDRS chart (Precision Vision, Woodstock, IL, USA) at 3 m, with luminance set to 160 cd/m^2^ per manufacturer’s recommendation, and a logMAR acuity was calculated through letter scoring. Contour stereopsis (Worth’s third level of fusion) was measured with the Randot stereo test book (Stereo Optical, Chicago, IL, USA) at 40 cm, whose measurable range of binocular disparity extends from 400 to 20 arc sec. The Stereofly stereo test book (Stereo Optical, Chicago, IL, USA), whose maximum disparity is 3500 arc sec at 40 cm, was used to measure subjects who could not perceive stereopsis with the Randot stereo test book. Measurements of stereopsis were converted to log arc sec, with subjects reporting no stereopsis assigned an arbitrary value of 4 log arc sec. A cover test with prism neutralization was performed to assess ocular alignment. Additionally, all subjects underwent a comprehensive optometric examination performed by the first author.

### 2.7. Statistical Analysis

Nonparametric testing was used for analysis, as data did not fall into a normal distribution. To examine whether there were significant differences found depending on each of the testing conditions, Friedman’s test (a nonparametric equivalent of repeated measures ANOVA) was used. The Wilcoxon signed-rank test was then used to conduct pair comparison testing with the Bonferroni correction factor applied. To investigate the correlation between the depths of suppression measured with the Synoptophore versus the Worth 4 Dot, Kendall’s tau-b correlation coefficient was calculated, with the Worth 4 Dot data treated as an ordinal variable. To examine whether there was a significant difference in the depth of suppression depending on amblyopic sub-group, the nonparametric Mann–Whitney U testing was used. To investigate the correlation between depth of suppression and the other clinical functions of visual acuity and stereoacuity, Kendall’s tau-b correlation coefficient was utilized again, this time with all variables treated as continuous. Figures throughout Section 3 have significant pair comparisons marked with a bracket and asterisk(s) using the following convention: significance ≤ 0.05 is marked with one asterisk, significance ≤ 0.01 is marked with two asterisks, and significance ≤ 0.001 is marked with three asterisks.

## 3. Results

### 3.1. Target Analysis

Measures on the Synoptophore and with the Worth 4 Dot were completed on each subject under multiple testing conditions. For depths of suppression measured by the Synoptophore, the six different pairs of slide targets previously discussed (three first-degree targets of progressively smaller size and three second-degree targets of progressively smaller size) created six different testing conditions. For depths of suppression measured by the Worth 4 Dot, the different testing distances and room lighting conditions combined to create four different testing conditions. This analysis was conducted to examine whether specific testing conditions yielded a more sensitive measure, with the plan to further investigate sub-group analysis for the sensitive measures identified by each test.

### 3.2. Synoptophore—Depth of Suppression

**Figure 3:**
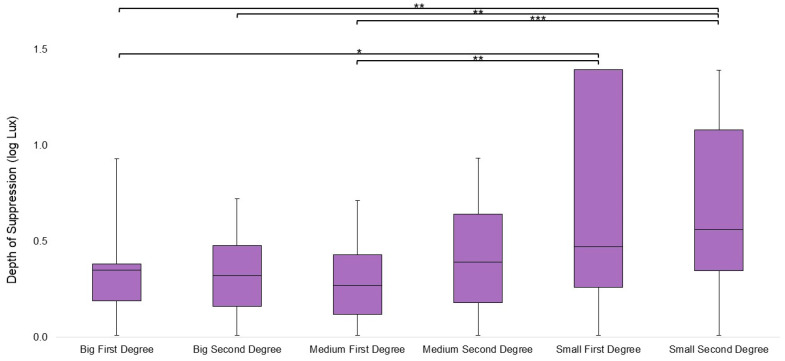
Suppression by the Synoptophore target condition. Shaded boxes represent the interquartile range, and whiskers extend to the maximums and minimums. Larger targets are plotted on the left side of the graph, with the smallest targets on the right. Significance ≤ 0.05 is marked with one asterisk, significance ≤ 0.01 is marked with two asterisks, and significance ≤ 0.001 is marked with three asterisks.

For the Synoptophore targets, an overall significant difference in the suppression measured by the different targets was found (χ^2^_5,26_ = 25.538, *p* < 0.001). Pair comparisons found a significant difference between the small first-degree target and the following two targets: big first-degree (*p* = 0.013) and medium first-degree (*p* = 0.004). There was also a significant difference between the small second-degree target and the following three targets: big first-degree (*p* = 0.005), big second-degree (*p* = 0.007), and medium first-degree (*p* < 0.001). In all instances, the smaller targets resulted in a higher measure of suppression than the bigger targets, as seen in Figure 3.

### 3.3. Worth 4 Dot—Depth of Suppression

**Figure 4:**
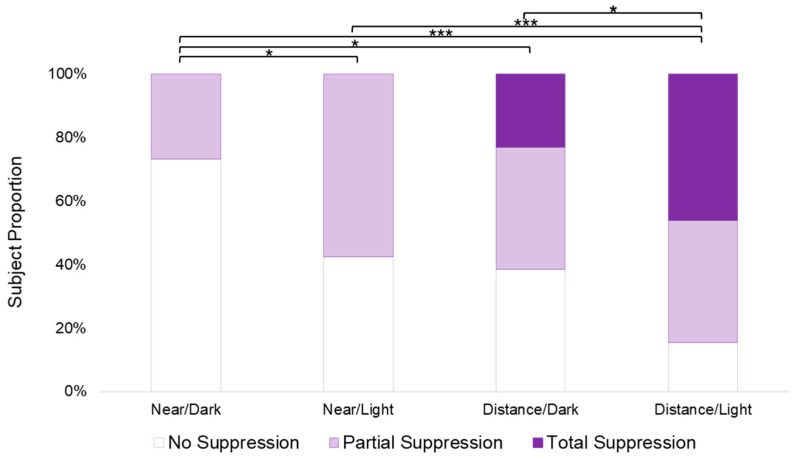
Suppression by the Worth 4 Dot condition. For each condition, the proportion of subjects with a “no suppression” response are represented by the white portion of each bar, the proportion of subjects with a “partial suppression” response are represented by the light grey portion of each bar, and the proportion of subjects with a “total” suppression response are represented by the dark grey portion of each bar. Significance ≤ 0.05 is marked with one asterisk and significance ≤ 0.001 is marked with three asterisks.

For the Worth 4 Dot test, an overall significant difference in the suppression measured by the different testing conditions was found (χ^2^_3,26_ = 39.020, *p* < 0.001). Except for the distance/dark and near/light comparison (*p* = 0.354), pair comparisons found a significant difference between all testing conditions, as seen in Figure 4 (near/dark vs. near/light: *p* = 0.03; near/dark vs. distance/dark: *p* = 0.006; near/dark vs. distance/light: *p* < 0.001; near/light vs. distance/light: *p* < 0.001; distance/dark vs. distance/light: *p* = 0.03). In all cases, the near/dark condition revealed less suppression than the other three testing conditions as a smaller proportion of subjects reported any suppression; the majority of subjects reported no suppression. The distance/light condition revealed more suppression than the other three testing conditions, with over 40% of subjects reporting total suppression of the amblyopic eye, and only 15% of subjects reporting no suppression.

### 3.4. Correlations between Tests

Two target conditions for each instrument were chosen to examine the relationship between the two instruments. For the Synoptophore, the targets of choice were the small first-degree and small second-degree targets as they revealed the greatest range of suppression measured out of all of the Synoptophore targets. For the Worth 4 Dot, both distance conditions (light and dark) were chosen. This was for two reasons: both showed more range of suppression measured as compared to at least one other Worth 4 Dot condition, and because both were conducted at a distance, the habitual ocular alignment of the subjects would better match the Synoptophore, which places its targets at optical infinity (simulated distance) through the use of lenses.

**Figure 5:**
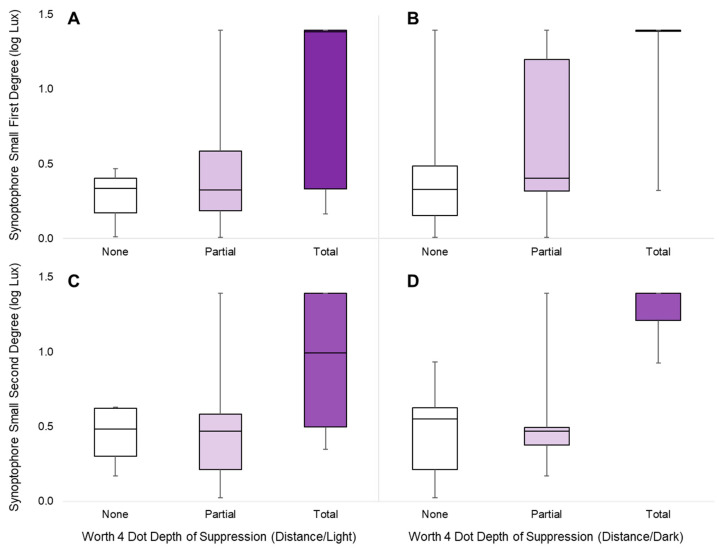
Depth of suppression with small Synoptophore targets correlated with Worth 4 Dot at a distance. Shaded boxes represent the interquartile range, and whiskers extend to the maximums and minimums. A positive correlation was observed between all pairs: small first-degree targets and distance Worth 4 Dot in light (**A**) and dark (**B**), and small second-degree targets and distance Worth 4 Dot in light (**C**) and dark (**D**).

A significant correlation was found for all comparisons and is illustrated in Figure 5. The depth of suppression measured with the small first-degree Synoptophore target was correlated with both Worth 4 Dot distance/light and distance/near (*r_Τ_* = 0.434, *p* = 0.009 for distance/light and *r_Τ_* = 0.345, *p* = 0.036 for distance/dark). This held true for the depth of suppression measured with the small second-degree target, as this measure was also correlated with both distance Worth 4 Dot lightning conditions (*r_Τ_* = 0.378, *p* = 0.021 for distance/light and *r_Τ_* = 0.462, *p* = 0.004 for distance/dark).

### 3.5. Sub-Group Analysis

**Figure 6:**
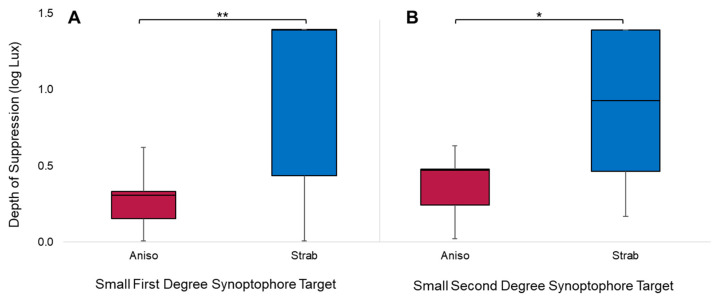
Depth of suppression with small Synoptophore targets in the amblyopia sub-group. Shaded boxes represent the interquartile range, and whiskers extend to the maximums and minimums. Higher depths of suppression were observed in strabismic amblyopes for both the small first-degree target (**A**) and the small second-degree target (**B**). Significance ≤ 0.05 is marked with one asterisk and significance ≤ 0.01 is marked with two asterisks.

As defined in the Methods section, two groups were determined for analyses: anisometropic amblyopes (“Aniso”) and strabismic amblyopes (“Strab”). Based on the previous target analysis section, the following conditions were considered for sub-group analysis: small first-degree and small second-degree targets for the Synoptophore depth of suppression, as these targets are more revealing of suppression. For the first-degree Synoptophore targets, there was a significant difference in the depth of suppression exhibited by the two sub-groups (*U* = 19, *z* = −3.266, *p* < 0.001). The median depth of suppression was higher in strabismic amblyopes (1.39 log Lux) than anisometropic amblyopes (0.31 log Lux). For the second-degree Synoptophore targets, there was also a significant difference in the depth of suppression exhibited by the two sub-groups (*U* = 34.5, *z* = −2.410, *p* = 0.016). As with the first-degree targets, the median depth of suppression was higher in strabismic amblyopes (0.93 log Lux) than anisometropic amblyopes (0.47 log Lux). These differences are illustrated in Figure 6.

### 3.6. Relationships with Other Visual Functions

**Figure 7:**
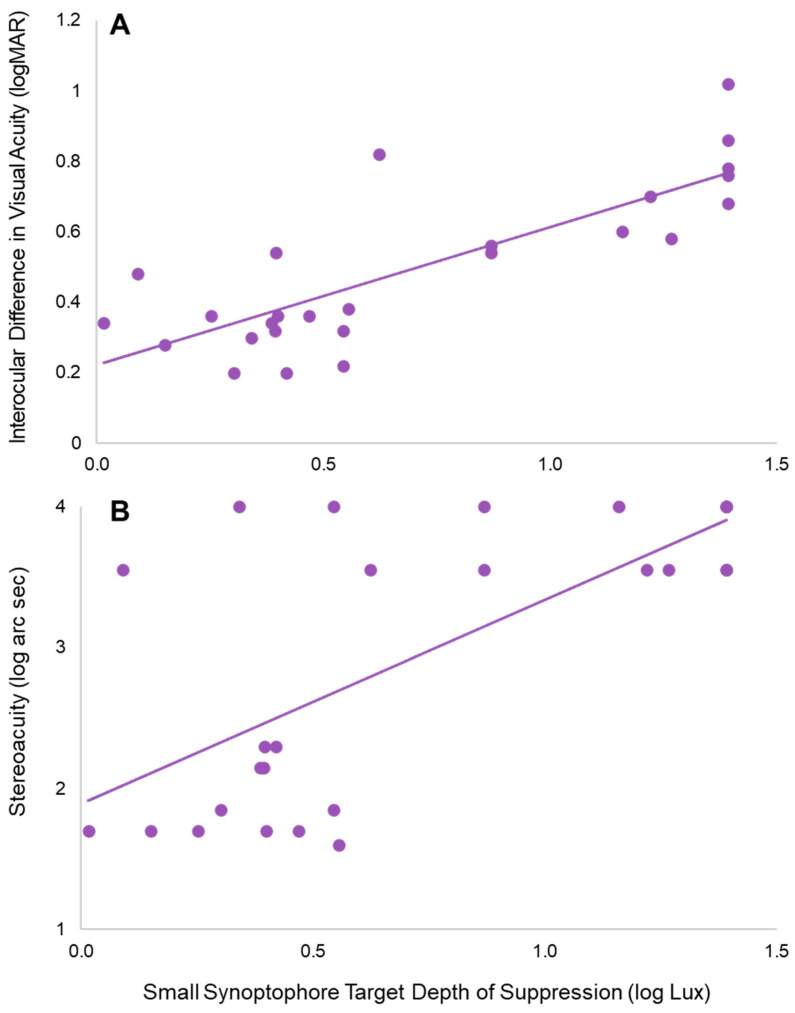
Small Synoptophore target depth of suppression correlated with interocular difference in visual acuity (**A**) and stereoacuity (**B**).

To compare the depth of suppression quantified by the Synoptophore to other measures of visual functions, the depths of suppression from the small Synoptophore targets were averaged together to best represent one overall metric for the Synoptophore. These two were chosen, as they were both, individually, sensitive targets for measuring suppression. By averaging them together, both of the foundational levels of fusion according to Worth (first- and second-degree) were taken into account when describing the holistic suppression of the individual subject. To determine whether this average depth of suppression from the small Synoptophore targets was associated with other clinical functions, depth of suppression was compared to interocular difference in visual acuity (logMAR) and stereoacuity (log arc sec). These comparisons are depicted in Figure 7 and reveal a significantly positive correlation between small target depth of suppression and interocular difference in visual acuity (*r_Τ_* = 0.604, *p* < 0.001) and small target depth of suppression and stereoacuity (*r_Τ_* = 0.488, *p* = 0.001). As depth of suppression increased, logMAR visual acuity increased, representing a poorer visual acuity. Similarly, as depth of suppression increased, log stereoacuity increased, representing worse stereopsis.

## 4. Discussion

The Synoptophore is commercially available to clinicians and is used to evaluate the more complex facets of the binocular visual system. It is a time-tested clinical instrument that has been used by clinicians for over a century. In addition to the instrument itself, all target stimuli used for this study were manufactured by Haag-Streit and, as such, readily provide for the normative standard to evaluate data obtained among different clinics. However, there is little research on the specifications of the instrumentation outside of the manual provided by the manufacturer, which lacks details about the illuminance output at each setting. One of our goals was to extend the utility of the Synoptophore by quantifying the illuminance levels to help guide clinicians in their use of the instrument for precise quantitative measures of binocular functions. 

Our analysis of the Synoptophore’s illumination system revealed a mostly log-linear progression between integer markings. Therefore, with our particular model of the instrument, if a patient has a two integer difference between the eyes, then there is an approximately 0.3 log unit difference between the eyes; twice the brightness is needed to overcome the interocular suppression. We are, of course, cognizant of the likelihood that not all models of Synoptophore would measure the same illuminance values. For instance, the light bulbs’ brightness changes with instrument age, and replacement light bulbs or rheostats might have different specifications. However, these hurdles could be mitigated by maintaining the Synoptophore with the factory-supplied parts. This will likely ensure the log-linearity of the illuminance outputs, making the quantification of suppression straightforward.

In our study, we measured suppression magnitudes with the targets provided by the manufacturer, as in standard practice, with a goal of identifying which target elicited the greatest suppression in our subjects. These targets differed in type (first- vs. second-degree fusion) and size (stimulating varying retinal eccentricity). We found the general trend that the small target sizes elicited the greatest depth of suppression. With the Synoptophore, this was achieved with the smallest angular-sized targets for each type (soldier size: 2.5 × 0.5°; rabbit size: 2.5 × 2.5°). With the Worth 4 Dot, this was achieved when the target was viewed at a 3 m distance (diamond size: 0.668 × 0.668°). This corresponds with the clinical notion of central suppression versus peripheral suppression. It has been documented that suppression characteristics can be altered depending on retinal location [8,16,33,34]. In general, targets involving the central part of the retina, particularly the fovea and the retinal location where the object of regard falls, in cases of strabismus, elicit suppression more frequently/excessively than more peripheral targets. As the target sizes decreased in our study, they progressively stimulated the foveal region of each eye relative to the more peripheral retinal regions. When measured in progression, as in our study, the depth of suppression for each target type and size provided extensive information about an individual’s sensory deficits. However, by identifying the target(s) that elicited the most suppression, clinicians seeking to extrapolate depth of suppression information through minimal amounts of measurement should consider testing with small first- and second-degree Synoptophore targets and Worth 4 Dot at a distance, as the patient response with this target will best indicate the maximum amount of suppression they must overcome.

The significant correlation between these small Synoptophore targets and the Worth 4 Dot at a distance is not surprising, especially considering the discussion of central suppression above. By correlating our measures from the Synoptophore with the Worth 4 Dot findings for each subject, we are able to validate our protocol with the Synoptophore through comparison to a widely used clinical tool. Qualitatively describing suppression with the Worth 4 Dot as none, partial, and total also extended the usefulness of this clinical tool by differentiating presumed normal responses (four dots are seen) with more nuance (e.g., unequal brightness or predominance of the fellow eye for the commonly seen dot). Even with this extra level of differentiation, there was still more sensitivity seen with the Synoptophore measures; as seen in Figure 5, when subjects responded with no suppression with Worth 4 Dot, they reported suppression with the Synoptophore, unveiling suppression that was unmeasurable by the former test.

The subjects in our study were adults who had previously been treated for amblyopia. They were classified as strabismic or non-strabismic (anisometropic) based on the suspected origin of their amblyopia. We found the overall depth of suppression between these two groups to be different. Namely, the strabismic subjects demonstrated an average depth of suppression that was two to three times that of non-strabismic subjects. This finding of a larger depth of suppression in the strabismic group is consistent with most basic psychophysical observations of more compromised sensorimotor functions. For example, McKee et al. (2003) found that non-binocular amblyopes (those with strabismus) tended to have worse optotype acuity and Vernier acuity, for a given level of grating acuity, than those with residual binocular function [7]. In another study, McKee et al. (2016) found that strabismic amblyopes had longer saccadic latency than non-strabismic amblyopes [35].

There was a strong correlation between suppression magnitudes and clinical measures; as suppression increased, performance on clinical tests decreased. Subjects with greater depths of suppression had greater interocular difference in visual acuity and poorer stereoacuity than subjects with a lesser depth of suppression. Correlations between suppression and clinical measures of visual acuity and stereoacuity have also been reported previously [36,37]. The observation of reduced stereopsis is not unique to amblyopia, as we have shown the same relationship in subjects with clinically normal vision. Specifically, we showed that subjects with higher magnitudes of sensory eye dominance, due to a larger depth of suppression, had a higher stereo threshold and stereo response time [15,16,17]. In finding that depth of suppression measures from the Synoptophore endorse previously found relationships between other clinical measures and suppression, it supports the use of the Synoptophore as a clinical instrument to quantify suppression in addition to other newly proposed technologies [22,23], with the added benefit that many working in binocular vision clinics already have access to it.

The goal of our investigation into the Synoptophore was to explore if use of the device could be extended to provide a quantitative measure of suppression in amblyopia that can be utilized by clinicians. We found the answer in the affirmative and also showed, in using a quantifiable clinical instrument, that the extent of suppression is correlated to deficits in other visual functions. We suggest that measuring the depth of suppression could better diagnose an individual’s extent of amblyopia and then be used as another metric to help guide treatment. Additionally, monitoring the depth of suppression during treatment may be more meaningful than a monocular measure, such as visual acuity, as it could better reflect the excessive inhibition in amblyopia. While monocular measures are important, binocular measures more directly address the causative nature of amblyopia, a binocular imbalance during vision development.

## Figures and Tables

**Table 1:** Clinical characteristics of subjects. FE = fellow eye; VA = visual acuity (logMAR); AE = amblyopic eye; IOD = interocular difference (logMAR); EDS = equivalent dioptric sphere; DCT = distance cover test (prism diopters); XP = exophoria; EP = esophoria; XT = exotropia; ET = esotropia. Stereopsis measurements are reported in log arc sec.

Group	ID	Age	FE VA	AE VA	IOD VA	FE EDS	AE EDS	DCT	Stereo
Aniso	A1	29	−0.04	0.24	0.28	−3.38	+3.25	6 XP	1.70
	A2	26	0.06	0.26	0.20	−3.50	+5.00	8 XP	1.70
	A3	21	−0.08	0.46	0.54	+1.50	+4.75	4 EP	2.30
	A4	30	−0.18	0.02	0.20	+0.88	+3.50	1 EP	1.85
	A5	26	−0.16	0.16	0.32	Plano	+5.38	4 XP	2.15
	A6	41	−0.12	0.26	0.34	+0.63	+4.13	2 XP	1.70
	A7	26	0.00	0.36	0.36	−0.25	+2.88	2 XP	1.70
	A8	26	−0.06	0.26	0.32	+0.13	+1.88	16 XP	1.85
	A9	28	−0.14	0.20	0.34	−0.13	+1.50	2 XP	2.15
	A10	30	0.06	0.42	0.36	−0.13	+1.25	4 XP	1.70
Strab	S1	25	−0.18	0.30	0.48	+0.75	+1.13	8 XT	3.55
	S2	29	−0.20	0.02	0.22	+2.88	+4.25	16 ET	4
	S3	22	−0.10	0.54	0.64	+2.38	+4.50	8 ET	4
	S4	23	0.02	0.56	0.54	−1.50	+1.63	20 XT	4
	S5	31	−0.14	0.24	0.38	−0.75	−0.88	16 ET	1.60
	S6	29	−0.20	0.00	0.20	+1.38	+2.63	12 ET	2.30
	S7	32	−0.26	0.04	0.30	+2.88	+3.25	8 XT	4
	S8	24	−0.14	0.68	0.82	+0.13	+2.50	2 ET	3.55
	S9	27	−0.08	0.52	0.60	+4.25	+4.75	10 XT	4
	S10	19	−0.08	0.68	0.76	+3.75	+8.00	2 ET	3.55
	S11	20	−0.08	0.48	0.56	+1.50	+3.50	2 ET	3.55
	S12	40	−0.24	0.78	1.02	+2.75	+5.63	2 ET	3.55
	S13	36	−0.04	0.74	0.78	+0.13	+0.88	6 ET	4
	S14	28	−0.18	0.68	0.86	+1.38	+3.00	6 ET	4
	S15	26	−0.04	0.66	0.70	−1.13	−0.88	4 ET	3.55
	S16	22	0.04	0.62	0.58	+5.50	+6.00	4 ET	3.55

**Table 2:** Categorization of suppression status with Worth 4 Dot testing.

No Suppression Equal perception of targets seen by the fellow and amblyopic eye (including diplopic response)
Partial Suppression One or more of the following perceptions: Unequal brightness: Targets of the amblyopic eye perceived as dimmer than fellow eye’s targets Intermittent suppression: Targets of the amblyopic eye only perceived some of the time Partial perception: Targets of the amblyopic eye not all visible (e.g., a left eye amblyope only appreciating one green dot present) Predominance of the fellow eye: White dot target perceived to be the color of the fellow eye’s filter
Total Suppression No perception of the amblyopic eye’s targets

## Data Availability

The data presented in this study are presently not available in a managed depository database but are available upon request.
